# A postmenopausal woman presenting with Ekbom syndrome associated with recurrent depressive disorder: a case report

**DOI:** 10.1186/1757-1626-1-54

**Published:** 2008-07-22

**Authors:** Courtney Mahler, Glenda MacQueen, Zainab Samaan

**Affiliations:** 1 McMaster University, 1280 Main St. West, Hamilton, Ontario, L8S 4L8, Canada; 2 St. Joseph's Healthcare, Dept. of Psychiatry and Behavioural Neurosciences, Centre for Mountain Health Services, Mood Disorders Program, 100 West 5th St, Hamilton, ON, L8N 3K7, Canada

## Abstract

**Background:**

Ekbom syndrome is a rare psychiatric disorder that can manifest as a delusion, overvalued idea or hallucination of parasitic infestations. It is more prevalent in postmenopausal women and patients are usually seeking dermatology rather than psychiatry consultation for their symptoms.

**Case presentation:**

We present a case of Ekbom syndrome associated with recurrent depressive disorder in an elderly patient. The patient presented with tactile hallucinations of insects crawling just under her skin. These hallucinations resolved with Mirtazepine and electroconvulsive therapy treatment in the absence of an antipsychotic pharmacological agent.

**Conclusion:**

This case report highlights the presence of a rare psychiatric presentation of Ekbom syndrome within the context of depression. The majority of such cases will not be seen by psychiatrists but by dermatologists. Therefore collaborative consultations between dermatologists and psychiatrists of patients presenting with symptoms of Ekbom are essential for the identification and management of such cases. The case also takes a look at possible aetiologies and the importance of descriptive psychopathology in distinguishing psychotic symptoms in depressive disorder.

## Background

Ekbom syndrome is a rare psychiatric disorder that usually presents to dermatology clinics. The literature is scant in systematic research into this interesting syndrome. It was first methodically described in a case series by Karl-Axel Ekbom in 1938 where a delusion of parasitic infestations associated with tactile hallucinations as well as skin manifestations were present in postmenopausal women[1]. The American Psychiatric Association described the condition in DSM IV as a delusional disorder.

Ekbom syndrome is often described as a delusion of infestation, however, it can also take the form of a tactile hallucination or overvalued idea[2]. The patient feels, believes or considers that they are infested by parasites or insects[2,3]. When this syndrome is present, the patient may reject the idea that the condition is psychiatric and refuse treatment, causing the initial presentation to be at a dermatology clinic[4]. However, this condition is often present as a symptom in mood disorders or other psychiatric illnesses such as in schizophrenia[2]. Ekbom may also occur in concordance with general medical conditions such as cerebrovascular disease and senile dementia[5,6]. Patients presenting with this illness are often elderly females over the age of 50[7,8]. Skin lesions are usually absent and pruritus is often a presenting complaint[9].

We present a case of Ekbom syndrome in an elderly depressed patient.

## Case presentation

We report a case of a 77-year old woman who presented with recurrent depressive disorder. The onset of depression was in early middle age and the course of illness has been recurrent with more severe episodes in later years. The current episode of depression started three years prior to this admission. In addition to depressive symptoms, the patient described developing a sensation of vermin moving under her skin all over her body. This tactile hallucination presented intermittently for past 2 years, but became more persistent as the severity of depression increased. She also complained of pruritus but had not sought medical advice for this. Her medical history was notable for benign essential tremor, hypertension, hypercholesterolemia and carotid endarterctomy.

The current episode of depression had failed to respond to several antidepressants. The patient had extreme sensitivity to medications' side effects and a conservative approach was adopted with her. On this admission the rationale for an antipsychotic medication was reviewed with her but she was reluctant to believe that she had a psychotic disorder. She was treated with Mirtazepine 30 mg daily as well as 12 sessions of bilateral electroconvulsive therapy (ECT) without an antipsychotic agent and her depressive episode as well as tactile hallucinations resolved completely.

## Discussion

Since its description in 1938[1], little has been written about the psychopathology, nosology and organic bases of Ekbom syndrome. Ekbom can present as a delusion, hallucination or overvalued idea[2]. Our case demonstrated the controversial nomenclature of the disorder as a delusion where the primary presentation was a hallucination that was possibly followed by a secondary delusion. The overall female to male ratio is 2:1, however before the age of 50 it is 1:1 and after it is 3:1[7]. This may suggest a hormonal predisposition to the condition. Ekbom syndrome has been reported to co-occur with bipolar and psychotic disorders in addition to presenting as a primary disorder with the recommended treatment of antipsychotics [10]. Due to the rarity of this condition producing small sample sizes, potential causes or predispositions have been difficult to study. The majority of cases present to dermatology clinics and patients are often reluctant to see psychiatrists for their skin manifestations. Tactile hallucinations have been reported in other psychiatric disorders such as dementia[5], it is may be possible therefore to infer that, like dementia, Ekbom syndrome may have a biological predisposition. Given the various presentation of the syndrome, the treatment should be tailored based on the presenting psychopathology. Our case responded well to antidepressant and ECT treatment in the absence of antipsychotics.

## Conclusion

In this case of Ekbom syndrome and depression, tactile hallucinations responded to a combination of Mirtazepine and ECT in the absence of antipsychotic medication adding to the debate of whether this is an isolated psychotic disorder or a unique psychosomatic pathology of postmenopausal women triggered by the changes in hormonal milieu. Since Ekbom syndrome associations with hormonal changes as well as organic brain disease such as dementia, it is logical to assume an organic aetiology. Systematic identification of this condition by dermatologists in consultation with psychiatrists would be helpful for estimating the frequency of this condition. It is also important to clarify whether Ekbom syndrome is restricted to certain patient profiles and whether it is responsive to particular treatment approaches.

## Consent

Written informed consent was obtained from the patient for publication of this case report. A copy of the written consent is available for review by the Editor-in-Chief of this journal.

## Competing interests

The authors declare that they have no competing interests.

## Authors' contributions

CM interviewed the patient and wrote 1^st ^draft. GM reviewed the clinical data, and contributed to the writing of the manuscript. ZS conceived the idea for the case report, obtained the clinical data, and wrote the final draft. All authors read and approved the final manuscript.
